# PIPLOM: prediction of exogenous peptide loading on major histocompatibility complex class I molecules

**DOI:** 10.1093/bioadv/vbaf037

**Published:** 2025-03-03

**Authors:** Florian Schmidt, Kanxing Wu, Lorenz Gerber, Florence Chioh Wen Jing, Daniel Carbajo, Glenn Wong Choon Lim, Melissa Wirawan, Christine Eng, Katja Fink, Daniel T MacLeod, Michael Fehlings, Andreas Wilm

**Affiliations:** ImmunoScape Pte Ltd, Singapore 228208, Singapore; ImmunoScape Pte Ltd, Singapore 228208, Singapore; ImmunoScape Pte Ltd, Singapore 228208, Singapore; ImmunoScape Pte Ltd, Singapore 228208, Singapore; ImmunoScape Pte Ltd, Singapore 228208, Singapore; ImmunoScape Pte Ltd, Singapore 228208, Singapore; ImmunoScape Pte Ltd, Singapore 228208, Singapore; ImmunoScape Pte Ltd, Singapore 228208, Singapore; ImmunoScape Pte Ltd, Singapore 228208, Singapore; ImmunoScape Inc, San Diego, CA 92121, United States; ImmunoScape Inc, San Diego, CA 92121, United States; ImmunoScape Pte Ltd, Singapore 228208, Singapore

## Abstract

**Summary:**

The exogenous, i.e. *in vitro*, loading of peptides onto major histocompatibility complex (MHC) class I molecules is a key step in many immunology-related experimental workflows. Here, we provide a machine learning solution, PIPLOM, which is specifically tailored to predict whether peptides can be loaded exogenously onto an MHC class I molecule. Benchmarking on 38 unseen epitopes with in-house ELISA (enzyme-linked immunosorbent assay) experiments showed that PIPLOM is outperforming well-established methods such as NETMHCpan-4.0 or MHCflurry, which are commonly used for the related task of predicting epitope HLA (human leukocyte antigen) haplotype specificity.

**Availability and implementation:**

Source code and data are available as Zenodo package 10.5281/zenodo.13771214. PIPLOM is available as a web service at https://piplom.immunoscape.com/.

## 1 Introduction

The major histocompatibility complex (MHC) class I presents antigenic peptides, i.e. peptides generated in the cytosol by the proteasome, primarily to CD8+ T cells, thereby enabling targeted immune responses (Van Kaer 2002). Predicting MHC class I restriction of T cell epitopes is a routine task in immunoinformatics supporting various applications such as neoantigen discovery, the development of advanced diagnostics, or the exploration of new avenues for cancer treatment (Lundegaard *et al.* 2010, Paul *et al.* 2020). Recent benchmarking showed that machine learning methods, such as NetMHCpan-4.0 (Jurtz *et al.* 2017) or MHCflurry 2.0 (O’Donnell *et al.* 2020), perform well in predicting MHC haplotype restriction relying on a combination of MHC binding and MHC ligand elution data (Paul *et al.* 2020) for model training.

**Figure 1.**
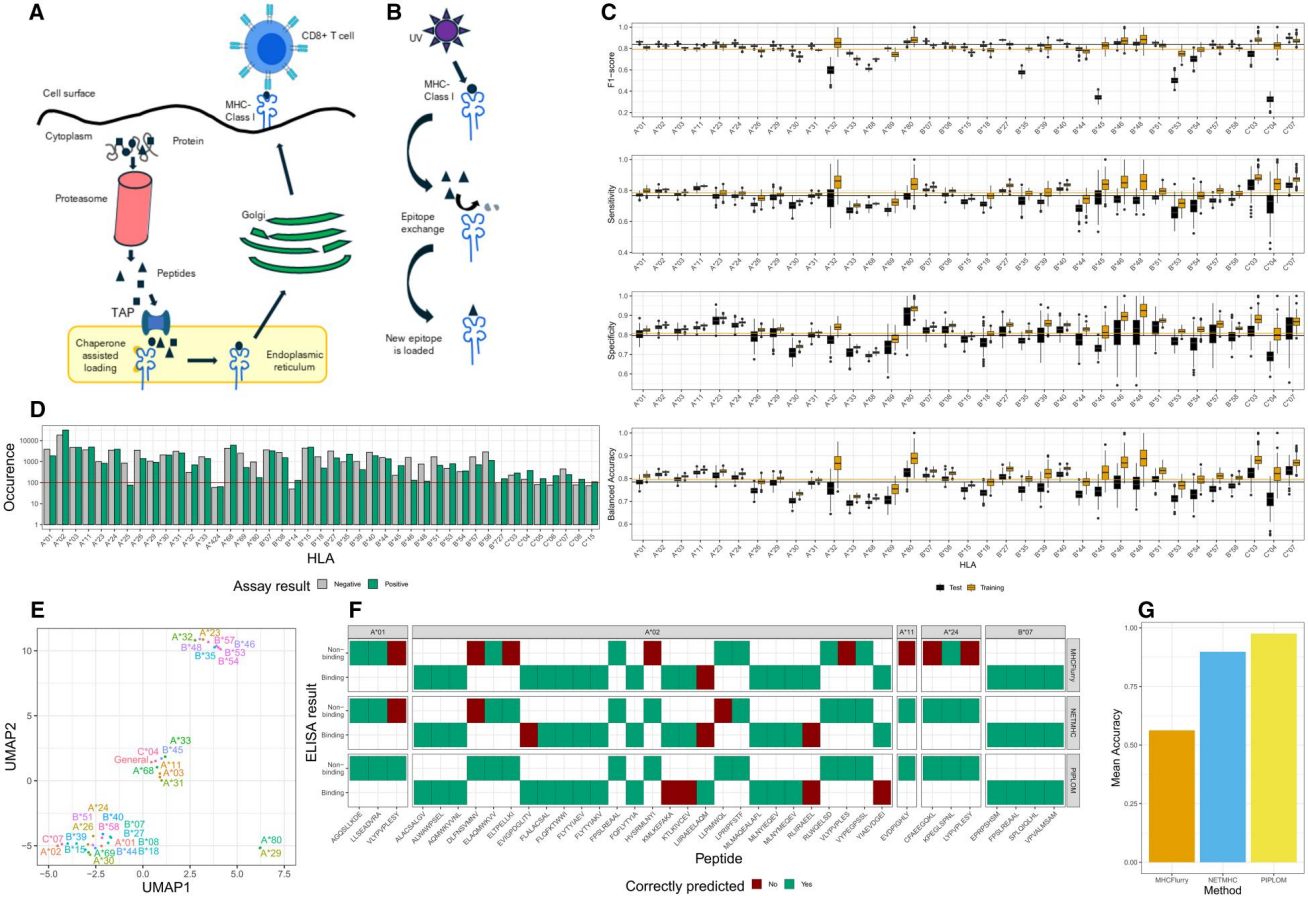
(A) Endogenous peptide loading is facilitated by chaperones within the endoplasmatic reticulum. The figure is adapted from Yewdell *et al.* (2003). (B) Exogenous, cell-free, peptide loading is based on peptide exchange triggered by UV exposure: First, the MHC is loaded with a high-affinity, UV sensitive peptide (containing a photo-cleavable bond) to stabilize the MHC in the peptide-bound conformation. Using UV light, the cleavable bond is broken, and the peptide dissociates from the MHC molecule. A new peptide is introduced and binds the now-empty MHC binding groove. (C) The performance of HLA-specific models is compared with the best performing HLA generic model (solid lines). (D) Available training data from IEDB per HLA and binding assay result. The threshold of 100 samples per class required for model training is indicated by the solid line. (E) UMAP (Uniform Manifold Approximation and Projection) visualization of regression coefficient vectors. (F) Comparison against NETMHCpan-4.0 and MHCflurry 2.0 on an in-house validation dataset consisting of 38 peptides across five HLAs. (G) Mean accuracy summarized per HLA for the validation data shown in panel (F).

While performing screening for antigen-specific T cells, we noticed that predictions of MHC haplotype restriction obtained with existing tools do not necessarily reflect whether epitopes can be successfully loaded onto the predicted HLA *in vitro*, as verified in ELISA assays (Sylvester-Hvid *et al.* 2002). We hypothesized that this is likely due to the incorporation of MHC ligand elution data into the training set used by the established models. These data points inform on peptide MHC haplotype pairing *in vivo* under physiological conditions (Bassani-Sternberg and Gfeller 2016). The natural loading process is shown in Fig. 1A. Antigens are cleaved into peptides via proteolysis before translocation into the endoplasmatic reticulum (ER) for loading onto MHC class I molecules, facilitated by chaperones and Tapasin (TAPBP) (Cresswell *et al.* 1999, Yewdell *et al.* 2003). The ER quality control ensures that only stably loaded peptide-MHC class I (pMHCI) molecules will be transported to the cell surface (Domnick *et al.* 2022). In contrast to that, exogenous setups lack this endogenous machinery. Instead, peptides are exchanged using, for instance, a ultraviolet (UV) or enzymatic cleavage method or are loaded onto empty MHC-I structures (Fig. 1B). Therefore, an epitope that can be loaded on an MHC molecule endogenously might not be loadable through an exogenous process.

To avoid wet lab experiments with unloaded epitopes, e.g. for T cell antigen profiling, we trained a machine learning model to predict whether a peptide can be loaded onto MHC class I molecules exogenously. We relied on MHC ligand binding assays from the Immune Epitope Database (IEDB) (Vita *et al.* 2019) as input, thus excluding potentially confounding ligand elution data. To our knowledge, no such method exists to date. The workflow of PIPLOM is visualized in Supplementary Fig. S1.

On an in-house validation set consisting of 38 unseen, i.e. not used for training, epitopes across five HLAs, we find that PIPLOM (mean accuracy: 0.970) performs better in predicting exogenous peptide loading compared to established state-of-the-art methods for the prediction of HLA haplotype specificity of peptides such as NetMHCpan-4.0 (mean accuracy: 0.896) and MHCflurry 2.0 (mean accuracy: 0.563). PIPLOM is publicly available as a web service at https://piplom.immunoscape.com/.

We note that the related problem of T cell receptor binding prediction is considerably more challenging, as in addition to the peptide MHC complex, the entire T cell receptor needs to be modeled too (Weber *et al.* 2024).

## 2 Materials and methods

### 2.1 IEDB data preprocessing

We downloaded data from 180 182 MHC binding assays restricted to class I MHCs from the IEDB (Vita *et al.* 2019) (14 May 2024). Binding assay results declared as “Positive-low,” “Positive-High,” “Positive-Intermediate,” and “Positive” were considered as a single class labeled as “Positive.” We removed MHC binding assay results with (i) not fully resolved HLA annotation, (ii) noncanonical amino acids occurring in the tested peptides or co-factors being present, or (iii) tested for an HLA that is linked to less than 100 positive and negative entries. Thus, we obtained a training data set of 175 484 data points covering 39 245 unique epitopes across 35 different HLAs.

### 2.2 Validation set and ELISA experiments

We performed ELISA experiments to generate an in-house validation set of 38 data points testing peptide loading of epitopes onto MHCs across five different HLAs (Supplementary Table S1). We focused on the commonly occurring HLA-A*01 and HLA-A*02 due to their global relevance, on HLA-A*11 and HLA-A*24 due to their prevalence in Asia, and on HLA-B*07 due to its prevalence in Europe.

ELISA is used here to detect stable pMHCI complexes consisting of HLA class I α-chain, a β2−microglobulin subunit (β2M), and the antigenic peptide. Specifically, an anti-β2M antibody is coated onto the bottom surface of wells in 96-well plates to capture pMHCI complexes via the β2M subunit. Intact and stable complexes were then detected with Avidin-Peroxidase that binds the biotinylated α–chain. Peptides that fail to load will result in the dissociation of the pMHCI complex and hence a lack of Avidin-Peroxidase binding detected. Inherent background for each HLA class was set against conditions where the UV-cleavable peptide was cleaved without the addition of a replacement/exchange peptide. This should result in largely dissociated MHCI complexes and a theoretical absence of avidin-HRP signals. Further details are provided in Supplementary Section S1.

### 2.3 Model description

#### 2.3.1 Physicochemical features

Physicochemical features of proteins are computed as described previously by Dubchak *et al.* (1993, 1995). Briefly, for any epitope, we computed 3 composition ($C=\{c_{1},c_{2},c_{3}\}$), 3 transition ($T=\{t_{1},t_{2},t_{3}\}$), and 15 distribution ($D=\{d_{11},\ldots ,d_{15},d_{21},\ldots ,d_{25},d_{31},\ldots ,d_{35}\}$) features with respect to seven amino acid-specific property groups: hydrophobicity, polarizability, polarity, van der Waals, charge, solvent, and structure (Supplementary Table S2). Further details are provided in Supplementary Section S2. Generally, due to the binning of the epitope sequence and/or length normalization during feature computation, PIPLOM features can be compared across epitopes with varying lengths.

#### 2.3.2 Model training and testing

We trained logistic regression classifiers with elastic net regularization using the glmnet R-package (4.1.7) (Friedman *et al.* 2010). The hyper-parameter α regulating the tradeoff between Lasso and Ridge regularization is fitted in a 10-fold Monte Carlo cross-validation (CV) procedure with a grid-search between 0.0 and 1.0 and a step-size of 0.01 using 20% of the data for testing and 80% for model fitting within a 6-fold inner cross validation.

Data bleeding, i.e. epitopes being shared between test and training, was avoided by the used sampling procedure. The entire model training process was repeated five times using different quality control-cutoffs t on the IEDB data with respect to the number of replicates for the binding assays per epitope $r_{e}$, with $r_{e}\geq t\in \{1,2,3,4,5\}$.

We utilized the above-described model training procedure to obtain four variations of models: (i) HLA agnostic, epitope length agnostic, (ii) HLA agnostic, epitope length specific, (iii) HLA-specific, epitope length agnostic, and (iv) HLA-specific, epitope length specific.

For each of the above variations, a model was only trained if the training set contained at least 50 positive and 50 negative samples.

Finally, for each HLA and epitope length combination, the best model was identified by comparing the CV errors among the tested model variants. The selected models were used for predictions on the in-house-generated validation set.

#### 2.3.3 PIPLOM web service

Trained models along with an R-based prediction script were installed in a Docker container that serves as a run-time image for a serverless compute function (Amazon AWS Lambda platform). The service can be accessed via https://piplom.immunoscape.com/. Models used by the web service will be updated in line with major updates of the IEDB.

### 2.4 Related methods for HLA prediction

#### 2.4.1 NETMHCpan-4.0

Using the NETMHCpan-4.0 webserver (Nielsen *et al.* 2003, Andreatta and Nielsen 2016), we obtained predictions for all peptides of the validation set. The column *BindLevel* was used to distinguish between binders and non-binders. Weak binders were considered as binders.

#### 2.4.2 MHCflurry 2.0

We installed MHCflurry 2.0 using pip as described by the authors and generated predictions for the validation set using the mhcflurry-predict command. We used the *mhcflurry-affinity-percentile* column in the generated output file with the suggested 2% threshold to distinguish binders from non-binders.

## 3 Results

### 3.1 PIPLOM accurately predicts exogenous peptide loading on MHC-class I

We trained four variations of models to predict exogenous peptide loading: (i) a model across HLA types and epitope length referred to as the general model; (ii) models across HLA types but with respect to epitope length; (iii) HLA-specific models across epitope length; and (iv) HLA and epitope length specific models.

The general model had a median F1 score of 0.837 in a 10-fold Monte Carlo CV (Fig. 1C). However, the CV performance of the general model showed a strong dependence on the epitope occurrence hyperparameter. We noticed a loss of generalization ability on the validation set with an increasing threshold (Supplementary Fig. S2A). This dependence is also present when models are trained separately for epitopes of different lengths, which also leads to a marginal performance improvement for epitopes of length 9 on the validation set (Supplementary Fig. S2B).

While the HLA-specific models perform on-par with the generic models for many HLAs in terms of F1-score, the HLA-specific models do outperform the generic model for several HLAs in terms of balanced accuracy in Specificity (Fig. 1C). Generally, the HLA-specific models performed best with the epitope occurrence hyper-parameter set to one, i.e. when all available data are considered for training (Supplementary Fig. S3A for training and Supplementary Fig. S3B for test error). Considering epitope length in the HLA-specific case did result in improved predictions for most HLAs (Supplementary Fig. S4). Interestingly, we did not find a correlation between the number of available training data points per HLA (Fig. 1D) and the mean performance of the HLA-aware models (ρ=0.096). This suggests two things: (i) for HLAs with poor prediction performance either the experimental data quality is not optimal and/or feature and model design cannot adequately capture the underlying binding mechanisms and (ii) rare HLAs, such as HLA-C*03 or HLA-C*07, for which only few data points exist, could still allow for a well-performing model to be trained.

A low-dimensional embedding of the regression coefficients reveals a distinct grouping of the HLA-specific models (Fig. 1E). This grouping partially reflects HLA supertypes (Sidney *et al.* 2008), e.g. HLA-A*31, HLA-A*11, HLA-A*03, HLA-A*33, and HLA-A*68 belonging to the A3 supertype are grouped next to each other. Interestingly, the HLA generic model is grouped with a subset of HLA-specific models, suggesting that the HLA-agnostic model is highly similar to a subset of the HLA-specific models, while others do learn a different signature.

As shown in Fig. 1F, PIPLOM achieves superior performance compared to the related methods NetMHCpan-4.0 (Jurtz *et al.* 2017) and MHCflurry 2.0 (O’Donnell *et al.* 2020) on an in-house validation set consisting of 38 peptides (Supplementary Table S1). In terms of mean accuracy across HLAs, PIPLOM achieved an accuracy of 0.970, while NETMHC-pan4 and MHCflurry 2.0 achieved values of 0.896 and 0.563, respectively (Fig. 1G). It is important to note that this comparison is not intended to simply showcase model superiority but to demonstrate the need for a separate model tailored for the specific use-case of predicting exogenous peptide loading, compared to the established HLA haplotype specificity problem addressed by NetMHCpan-4.0 and MHCflurry 2.0.

Specifically, both NETMHC-pan4 and MHCflurry 2.0 failed to accurately predict the loading of VLYPVPLESY on HLA-A*01 and DLFNSVMNV, as well as LIIRAEELAQM on HLA-A*02 (Fig. 1F). We note that the peptide FPSLREAAL is correctly predicted to be loadable on HLA-B*07 but not on HLA-A*02. This clearly highlights the need for HLA-aware modeling. Importantly, PIPLOM correctly predicted all non-binders included in the validation set. This highlights its usefulness in avoiding unnecessary wet lab experiments with unloaded peptides.

### 3.2 A web service for loading predictions

PIPLOM is accessible to the community as a web service and is available at https://piplom.immunoscape.com/ (Supplementary Fig. S5). As input, users have to provide one or multiple epitopes and the corresponding HLAs of interest (Supplementary Fig. S6). Loading predictions are presented for all queried epitopes both in terms of likelihood and as a class label (Supplementary Fig. S7).

## 4 Discussion and conclusion

We present PIPLOM, a machine learning model tailored to predict whether epitopes can be loaded onto MHC-class I molecules exogenously. This task is distinct from the related task of predicting the HLA haplotype specificity of peptides. The latter is addressed by state-of-the-art methods, such as NETMHC-pan4 and MHCflurry 2.0. Due to the lack of methods that specifically predict exogenous peptide loading and the relatedness of the two tasks, we benchmarked against these two methods. PIPLOM achieves superior performance on an in-house validation set despite relying on a logistic regression model. While conceptually simple, linear models provide computational efficiency, are easy to interpret, and are less prone to overfitting when trained on datasets of limited size or poor quality than more advanced nonlinear machine learning algorithms. Hence, we rely on logistic regression for this initial implementation of PIPLOM.

We hypothesize that the superior performance of PIPLOM is likely due to the tailored curation of the training dataset from IEDB, which excluded both ligand elution assays and predicted MHC binding affinities. By doing so, we allowed the model to learn signatures of exogenous binding assays based on synthesized peptides. These reflect the exogenous loading setup much more accurately than ligand elution assays and corresponding predicted binding affinities. Nonetheless, it is likely that the usage of more sophisticated machine learning models could boost model performance even further, especially in cases where HLA-specific models do not show a performance improvement over the general model.

A potential other explanation for the sub-optimal performance of HLA-specific models observed in some instances might be the nature of the experimental data used for training. Based on our in-house experience, interpreting and performing HLA binding assays is not equally simple and easy for every HLA type. Specifically, different levels of avidin-HRP background observed in each HLA class could reflect differences in the nonspecific binding of each dissociated class I α-chain even as a functional pMHCI complex is no longer present upon the loss of the UV-cleavable peptide. While ELISA is designed to detect the presence of an α-chain-β2M complex, it cannot differentiate between a functional Phi configuration from a nonfunctional one. Such nonspecific signals could potentially be mitigated by means such as (i) titrating the avidin-HRP amounts used for each HLA class or (ii) increasing or modifying wash conditions to remove noncomplexed α-chains and reduce their nonspecific retention. With the ELISA used for validation here, we observed a higher background and hence a narrower assay window for HLA-A*03 and HLA-A*24 and tried to partially mitigate the background noise by loading a fifth of the total amounts of soluble pMHC per well as compared with that used for HLA-A*02 and HLA-A*01. For these HLAs, interpretation of assay results can be complicated even after normalization against internal positive and negative controls. Therefore, suboptimal experimental data quality might make the prediction of certain HLAs more challenging. This could be easily addressed by adding in more high-quality, cross-validated data for model training.

In the future, it might be of interest to improve the accuracy of loading predictions by using ensemble methods such as boosting or bagging during model training. At the same time, adding custom weights to data points with respect to training data quality and replicate counts could help improve model generalizability and performance even further. In addition, especially if more high-quality training data are available, one could consider fitting nonlinear, e.g. random-forests, as well as deep learning methods instead of the logistic regression model. Furthermore, in addition to the physicochemical features considered here, a systematic evaluation of other feature computation strategies, e.g. k-mer counts or transformation using the AA-index (Kawashima and Kanehisa 2000), could be performed. Aside from methodological advancements, a workflow-based implementation of PIPLOM would also be desirable to simplify updating and deploying improved classifiers with new data releases on IEDB. We envision such an enhanced version of our method to be expanded to MHC-class II molecules and to be easily integrated into existing immunopeptidomics pipelines as well, for instance, by providing APIs for third-party access to the web service.

In its current form, PIPLOM showcases the need to specifically address the problem of exogenous peptide loading onto MHC class I molecules. It provides a first of its kind and easily accessible solution to this problem, enabling academic and industrial laboratories to streamline peptide screening panels and thus save experimental resources. Consequently, PIPLOM also aids in identifying cases where more complex assays are required to further characterize T cell responses against epitopes that can only be loaded endogenously. As such, PIPLOM would be relevant in any scenario that utilizes a cell-free, exogenous peptide loading system for soluble MHC class I. This can be in the form of a discovery or validation tool for antigen-specific T cell detection, such as multimeric soluble MHC class I monomer technologies. Also, vaccines that are engineered around MHC class I loaded with exogenous antigenic peptides in a cell-free context would benefit from PIPLOM models.

## Supplementary Material

vbaf037_Supplementary_Data

## Data Availability

Data and code used for model training and validation are available on Zenodo (10.5281/zenodo.13771214).
