# Correction to: PIPLOM: prediction of exogenous peptide loading on major histocompatibility complex class I molecules

**DOI:** 10.1093/bioadv/vbaf138

**Published:** 2025-06-27

This is a correction to: Florian Schmidt, Kanxing Wu, Lorenz Gerber, Florence Chioh Wen Jing, Daniel Carbajo, Glenn Wong Choon Lim, Melissa Wirawan, Christine Eng, Katja Fink, Daniel T MacLeod, Michael Fehlings, Andreas Wilm, PIPLOM: prediction of exogenous peptide loading on major histocompatibility complex class I molecules, *Bioinformatics Advances*, Volume 5, Issue 1, 2025, https://doi.org/10.1093/bioadv/vbaf037

The following change has been made to the originally published manuscript. The last name of the fifth co-author is emended to read: “Carbajo” instead of: “Pedrosa”.

