# Supplementary material for: PIPLOM: prediction of exogenous peptide loading on major histocompatibility complex class I molecules
Source: Bioinform Adv. 2025 Mar 3;5(1):vbaf037. doi: 10.1093/bioadv/vbaf037 (PMC11904885; doi:10.1093/bioadv/vbaf037)
Supplement: vbaf037_Supplementary_Data [file vbaf037_supplementary_data.pdf]

Supplementary Materials - PIPLOM: Prediction of In-vitro Peptide Loading on Major Histocompatibility complex Class 1 Molecules

1 In-house validation data

1.1 Summary

For several peptides of interest that had no MHC ligand binding data available on IEDB, we performed ELISA experiments and ran HLA specificity/loading predictions with NETMHCpan-4.0, MHCFlurry and PIPLOM, respectively (Supplementary Table 1). Experimental details on ELISA experiments are provided in the next section.

Peptide	HLA	ELISA readout	NETMHCpan-4.0	MHCFlurry	PIPLOM
**AGQSLLKDE**	A01	Negative	Negative	Negative	Negative
**LLSEADVRA**	A01	Negative	Negative	Negative	Negative
**VLYPVPLESY**	A01	Negative	Positive	Positive	Negative
**ALWAWPSEL**	A02	Positive	Positive	Positive	Positive
**FLQFKTWWI**	A02	Positive	Positive	Positive	Positive
**FPSLREAAL**	A02	Negative	Negative	Negative	Negative
**LLPIMWQL**	A02	Negative	Positive	Negative	Negative
**MLMAQEALAFL**	A02	Positive	Positive	Positive	Positive
**RLWQELSD**	A02	Negative	Negative	Negative	Negative
**VLYPVPLES**	A02	Negative	Negative	Positive	Negative
**VYPEGPSSL**	A02	Negative	Negative	Negative	Negative
**EVDPIGHLY**	A11	Negative	Negative	Positive	Negative
**LYPVPLESY**	A24	Negative	Negative	Positive	Negative
**EPRPSHSM**	B07	Positive	Positive	Positive	Positive
**FPSLREAAL**	B07	Positive	Positive	Positive	Positive
**SPLQIQLHL**	B07	Positive	Positive	Positive	Positive
**VPVALMSAM**	B07	Positive	Positive	Positive	Positive
**KPEGLSPNL**	A24	Negative	Negative	Negative	Negative
**CFAEEGQKL**	A24	Negative	Negative	Positive	Negative
**LPRIPFSTF**	A02	Negative	Negative	Negative	Negative
**ELTPELLKI**	A02	Negative	Negative	Positive	Negative
**KTLKIVCEV**	A02	Positive	Positive	Positive	Negative
**YIAEVDGEI**	A02	Positive	Positive	Positive	Negative
**LIIRAEELAQM**	A02	Positive	Negative	Negative	Negative
**RLIIRAEEL**	A02	Positive	Negative	Positive	Positive
**ELAQMWKVV**	A02	Negative	Negative	Negative	Negative
**AQMWKVVNL**	A02	Positive	Positive	Positive	Positive
**HVSRMLNYI**	A02	Negative	Negative	Positive	Negative
**DLFNSVMNV**	A02	Negative	Positive	Positive	Negative
**MLNYIEQEV**	A02	Positive	Positive	Positive	Positive
**FLYTYIAKV**	A02	Positive	Positive	Positive	Positive
**ALACSALGV**	A02	Positive	Positive	Positive	Positive
**EVIGPDGLITV**	A02	Positive	Negative	Positive	Positive
**MLNYMEQEV**	A02	Positive	Positive	Positive	Positive
**FQFLYTYIA**	A02	Positive	Positive	Positive	Positive
**FLALACSAL**	A02	Positive	Positive	Positive	Positive
**FLYTYIAEV**	A02	Positive	Positive	Positive	Positive
**KMLKEFAKA**	A02	Positive	Positive	Positive	Negative

Supplementary Table 1: Validation set. *Details on ELISA experiments to check on successful peptide loading for various peptides. NETMHCpan-4.0, MHCFlurry and PIPLOM results are provided as well with incorrect predictions highlighted in red.*

1.2 Experimental details on ELISA

Enzyme Linked Immunosorbent Assay was done to determine which peptide of interest can be loaded to form a stable HLA class I complex after the UV-cleavable peptide was degraded. The assay was done using the LEGEND MAX™ Flex-T™ Human Class I Peptide Exchange ELISA kit from BioLegend according to manufacturer's instructions with some modifications detailed below. All HLA monomers used were generated in-house.

Target peptides were first exchanged onto biotinylated HLA monomers stabilized with a UV-cleavable peptide. Briefly, HLA and target peptide were mixed in a U-bottom 96-well plate (Corning) at final concentrations of 100µg/ml and 50µM respectively. Plate was exposed to ultraviolet (UV) light in a UV Crosslinker (Analytik Jena) at maximum energy output (9999µJoules/cm^2) for 3 consecutive 5-min intervals. Plate was then sealed and mixtures incubated at 4ºC overnight for exchange to occur.

Next, we loaded the peptide-exchanged HLA class I monomers onto streptavidin-coated microwell plates. A01, A02 and B07 monomers were loaded at a final concentration of 5ng/ml per well while A03 and A24 monomers were loaded at 1ng/ml each. Finally, we detect intact HLA class I complex using Horseradish Peroxidase (HRP)-conjugated anti-human β2- microglobulin. HRP signals for each test peptide is benchmarked against a class-specific negative control (UV-only, no peptide exchanged) and positive control (peptide-HLAs with a functional TCR from in-house data). Any readout equal or below the respective negative controls is considered a negative outcome. Results reported for each peptide were from at least 3 technical replicates.

2 Physicochemical properties describing peptides

The universal properties of amino acids in the protein sequence are characterized using 3 descriptors: Composition, Transition and Distribution. Further details can be found in Dubchak, I., et al., Prediction of protein folding class using global description of amino acid sequence. Proc Natl Acad Sci U S A, 1995. 92(19): p. 8700-4.

2.1 Amino acid property groups

As part of the characterization, amino acids are assigned to distinct groups describing the properties with respect to hydrophobicity, polarizability, polarity, vdw, charge, solvent and structure.

AA	hydrophobicity	polarizability	polarity	vdw	charge	solvent	structure
R	1	3	3	3	1	2	1
K	1	3	3	3	1	2	1
E	1	2	3	2	3	2	1
D	1	1	3	1	3	2	3
Q	1	2	3	2	2	2	1
N	1	2	3	2	2	2	3
G	2	1	2	1	2	1	3
A	2	1	2	1	2	1	1
S	2	1	2	1	2	3	3
T	2	1	2	1	2	3	2
P	2	2	2	1	2	3	3
H	2	3	3	3	2	3	1
Y	2	3	1	3	2	3	2
C	3	2	1	1	2	1	2
V	3	2	1	2	2	1	2
L	3	2	1	2	2	1	1
I	3	2	1	2	2	1	2
M	3	3	1	3	2	3	1
F	3	3	1	3	2	1	2
W	3	3	1	3	2	1	2

Supplementary Table 2: Assignment of property group values to Amino Acids.

For the following examples, we use the peptide EKKYFAATCFEPLAA and consider the property group *charge*.

We obtain the annotation:

Sequence	E	K	K	Y	F	A	A	T	C	F	E	P	L	A	A
Charge	3	1	1	2	2	2	2	2	2	2	3	2	2	2	2
Index	1	2	3	4	5	6	7	8	9	10	11	12	13	14	15

Supplementary Table 3: *Property group assignment for the peptide EKKYFAATCFEPLAA and property charge*

2.2 Composition features

The composition feature C_g is the frequency of a particular amino acid property group g normalized by the length of sequence *N* computed according to:

$$C_g = \left(\frac{n_{g1}}{N}, \frac{n_{g2}}{N}, \frac{n_{g3}}{N}\right).$$

Given the amino acid sequence EKKYFAATCFEPLAA of length *N = 15* and property group *charge*, we obtain:

$$C_{charge} = (\frac{2}{15}, \frac{2}{15}, \frac{11}{15}).$$

2.3 Transition features

Transition features T_g capture the number of transitions of within values of a property group for neighbouring amino acids normalized by the total number of potential transitions.

$$T_g = \left(\frac{T_{g_1g_2} \times 100}{N-1}, \frac{T_{g_1g_3} \times 100}{N-1}, \frac{T_{g_2g_3} \times 100}{N-1}\right).$$

Given the amino acid sequence EKKYFAATCFEPLAA of length *N-1 = 14* and property group *charge*, we obtain:

$$T_{charge} = (\frac{1}{14}, \frac{1}{14}, \frac{2}{14}).$$

2.4 Distribution features

Distribution features D_g describe where the first, 25%, 50%, 75%, and 100% of the amino acids of a particular property group value g_i are located within the peptide sequence and is computed according to:

$$D_g = (D_{g1}, D_{g2}, D_{g3}),$$

$$D_{g_i} = \left(\frac{P_{g_i0}}{N}, \frac{P_{g_i25}}{N}, \frac{P_{g_i50}}{N}, \frac{P_{g_i75}}{N}, \frac{P_{g_i100}}{N}\right).$$

For the example sequence EKKYFAATCFEPLAA, we obtain:

$$D_{g_1} = \left(\frac{2}{15}, \frac{2}{15}, \frac{2}{15}, \frac{3}{15}, \frac{3}{15}\right),$$

$$D_{g_2} = \left(\frac{4}{15}, \frac{6}{15}, \frac{9}{15}, \frac{12}{15}, \frac{15}{15}\right),$$

$$D_{g_3} = \left(\frac{1}{15}, \frac{1}{15}, \frac{1}{15}, \frac{11}{15}, \frac{11}{15}\right).$$

3 Supplementary Figures

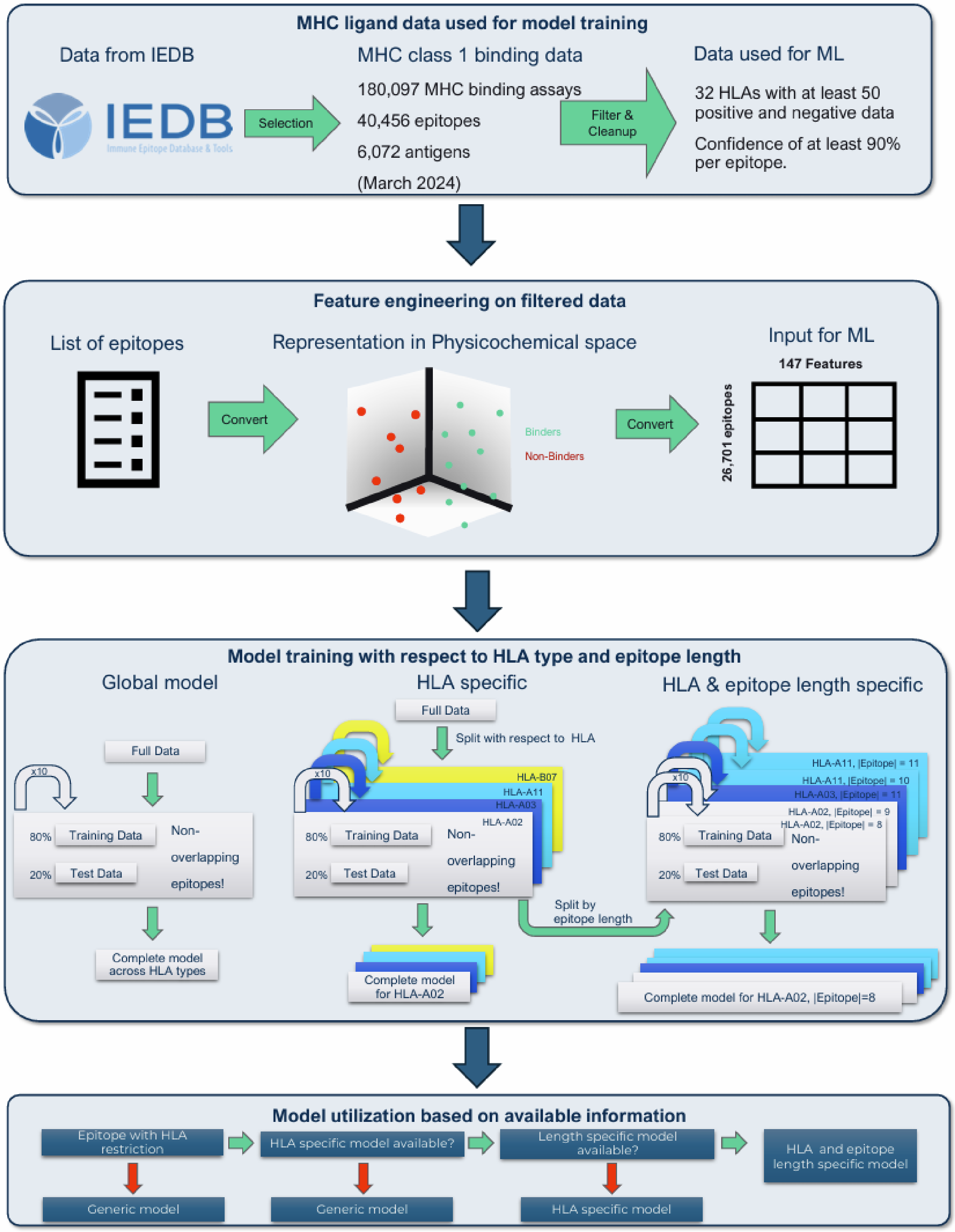

Supplementary Figure 1: Workflow chart. *MHC class I binding data is obtained from IEDB and pre-processed for quality. Next the remaining epitope sequences are converted into physicochemical space to generate the ML input matrix. Using this data, (1) the general model, (2) the HLA specific models and the (3) HLA as well as epitope length specific models. Depending on the available input and model performance, either one of the three models is used to process user queries.*

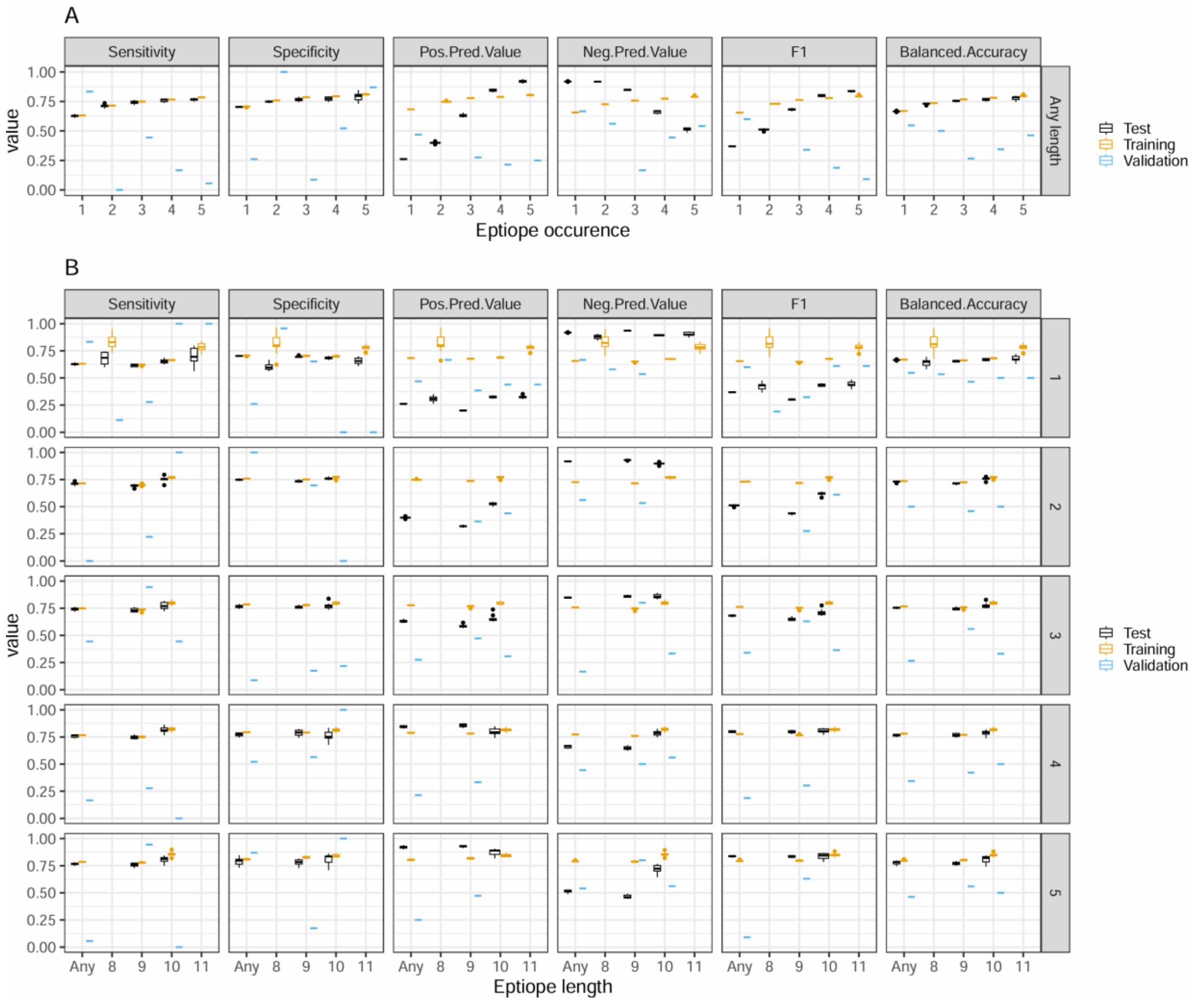

Supplementary Figure 2: Performance of the HLA agnostic models. A: *Performance of the HLA agnostic model on Test, Training and Validation sets with respect to different epitope occurrence cut-off used for model training.* ***B:*** *Same as A but restricting training and application of the HLA generic model to epitopes of a particular length.*

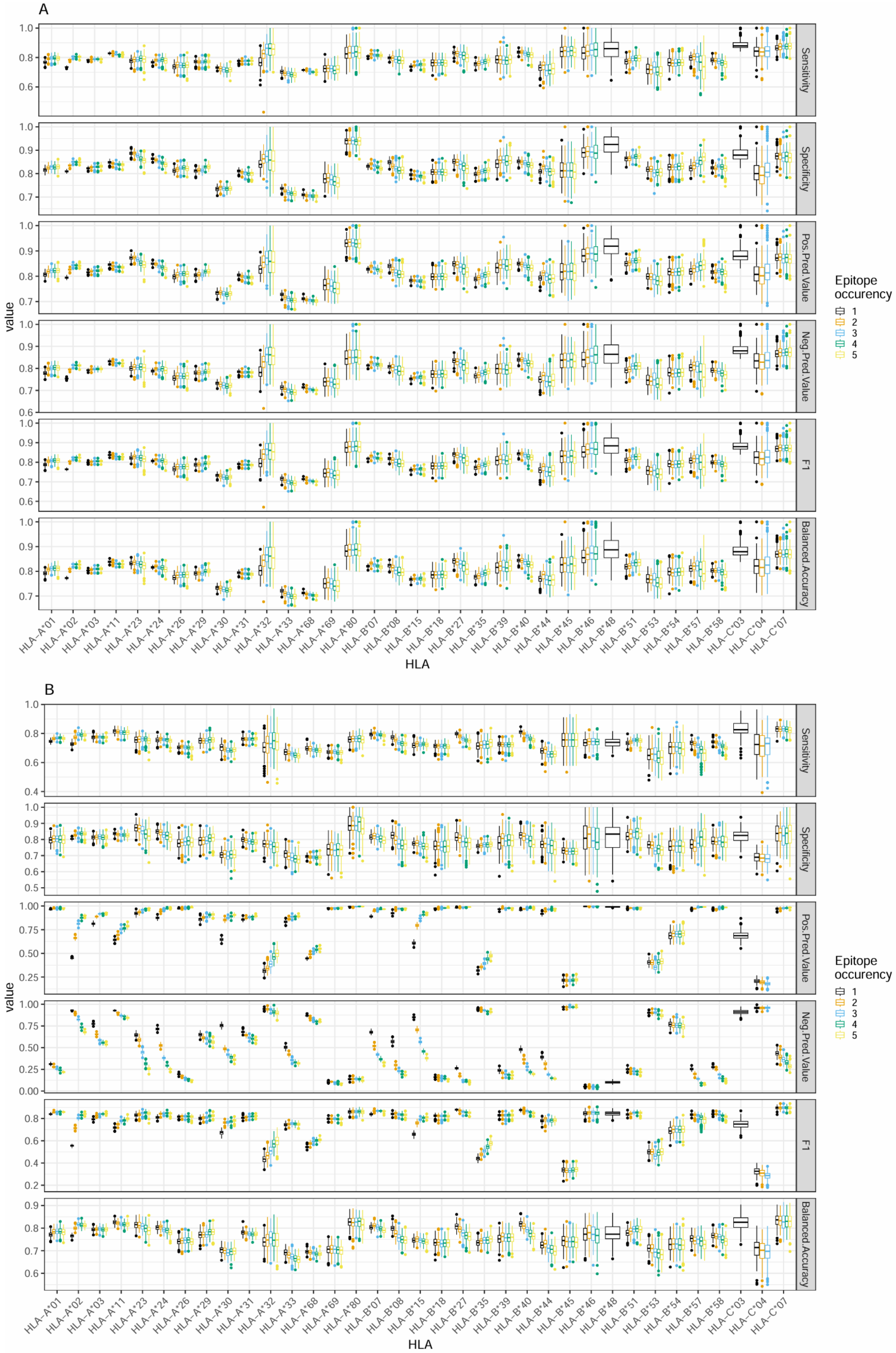

Supplementary Figure 3: Performance of the HLA specific models. A: *Training error estimated during 10-fold Monte-Carlo cross validation.* ***B:*** *Test error estimated using 10-fold Monte-Carlo cross validation.*

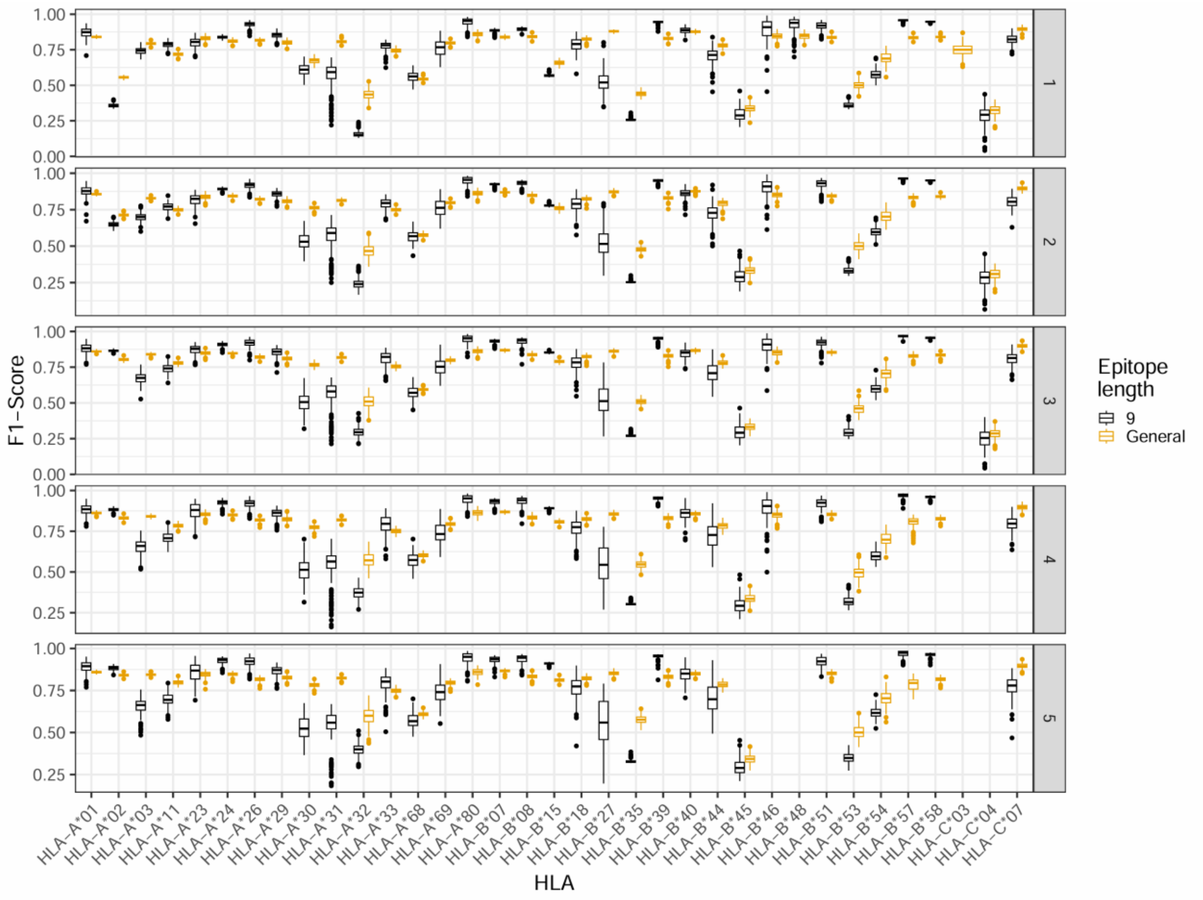

Supplementary Figure 4: *Comparison of HLA specific models to HLA and length specific models across different epitope occurrence cut-offs.*

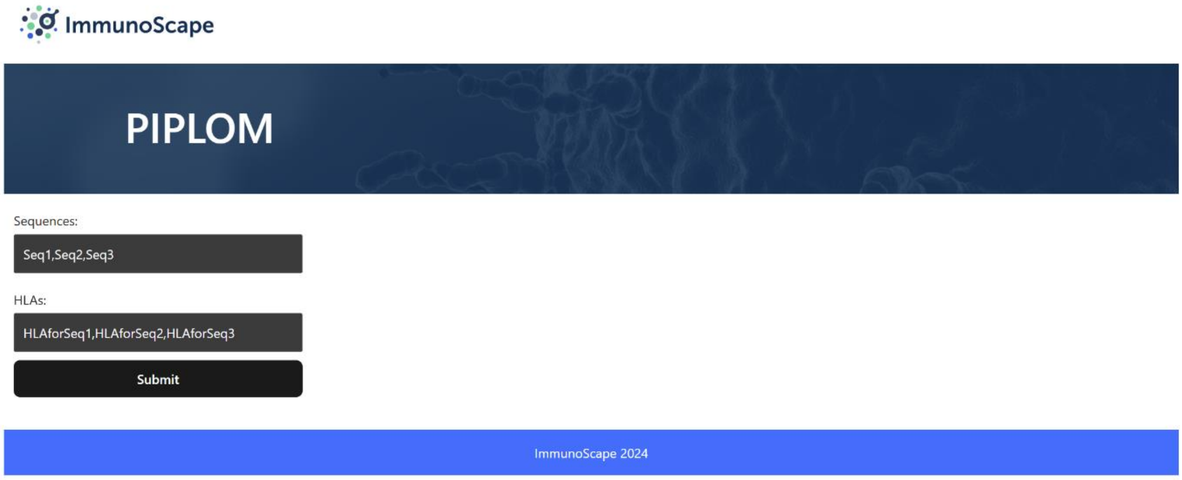

Supplementary Figure 5: *piplom.immunoscape.com overview*

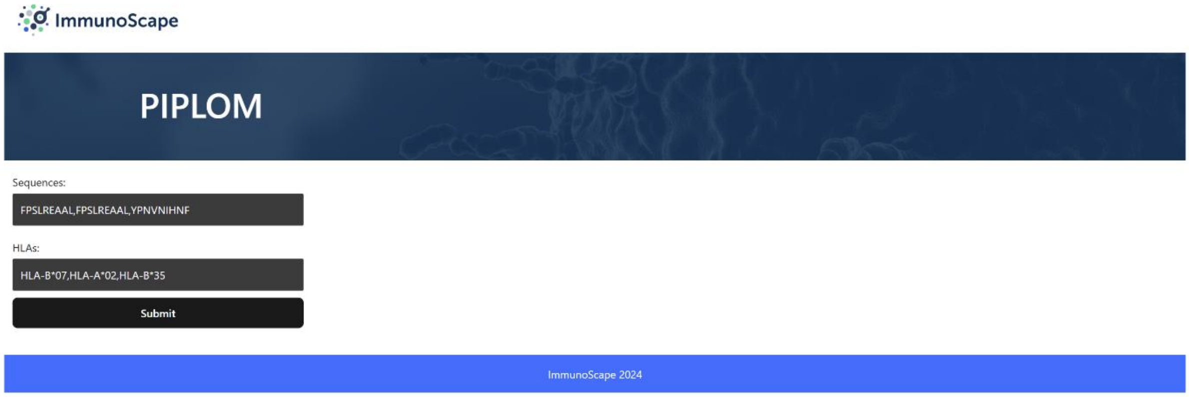

Supplementary Figure 6: *piplom.immunoscape.com with example input to obtain loading predictions for 2 peptides across 3 different HLAs*

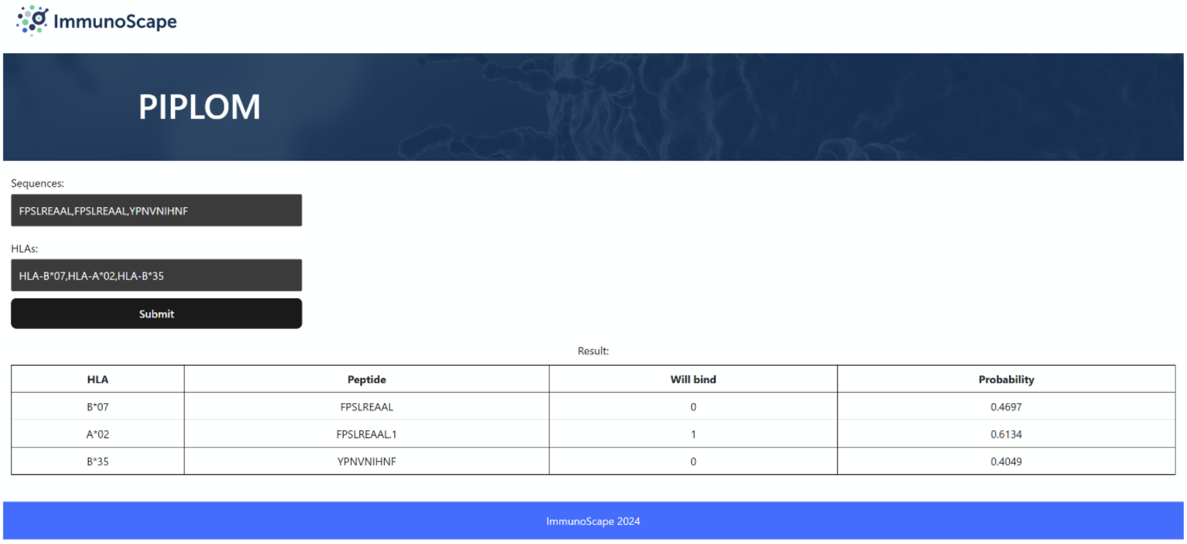

HLA	Peptide	Will bind	Probability
B*07	FPSLREAAL	0	0.4697
A*02	FPSLREAAL.1	1	0.6134
B*35	YPNVNIHNF	0	0.4049

Supplementary Figure 7: *piplom.immunoscape.com with example output obtaining loading predictions for 2 peptides across3 different HLAs*
